# Variants in the Dopamine-4-Receptor Gene Promoter Are Not Associated with Sensation Seeking in Skiers

**DOI:** 10.1371/journal.pone.0093521

**Published:** 2014-04-01

**Authors:** Cynthia J. Thomson, Amelia K. Rajala, Scott R. Carlson, Jim L. Rupert

**Affiliations:** 1 School of Kinesiology, University of British Columbia, Vancouver, British Columbia, Canada; 2 Department of Science, University of British Columbia, Vancouver, British Columbia, Canada; 3 Department of Psychology, University of Minnesota Duluth, Duluth, Minnesota, United States of America; Universtiy of Maryland Schoool of Medicine, United States of America

## Abstract

Sensation seeking is a personality trait that has been associated with disinhibited behaviours including substance use and gambling, but also with high-risk sport practices including skydiving, paragliding, and downhill skiing. Twin studies have shown that sensation seeking is moderately heritable, and candidate genes encoding components involved in dopaminergic transmission have been investigated as contributing to this type of behaviour. To determine whether variants in the regulatory regions of the dopamine-4-receptor gene (*DRD4*) influenced sport-specific sensation seeking, we analyzed five polymorphisms (−1106T/C, −906T/C, −809G/A, −291C/T, 120-bp duplication) in the promoter region of the gene in a cohort of skiers and snowboarders (*n* = 599) that represented a broad range of sensation seeking behaviours. We grouped subjects by genotype at each of the five loci and compared impulsive sensation seeking and domain-specific (skiing) sensation seeking between groups. There were no significant associations between genotype(s) and general or domain-specific sensation seeking in the skiers and snowboarders, suggesting that while *DRD4* has previously been implicated in sensation seeking, the promoter variants investigated in this study do not contribute to sensation seeking in this athlete population.

## Introduction

Downhill sports such as skiing and snowboarding are popular high-risk pastimes that often involve high speeds, hazardous terrain, and uncertain weather conditions and thus carry a potential for severe injury [1]. The personality trait, sensation seeking, has commonly been associated with high-risk sport participation [1], as well as with risky “socially deviant” activities such as gambling [2], binge drinking [3], and drug use [4]. While environmental factors likely influence attitudes towards, and behaviour in, high-risk sports, twin studies have shown sensation seeking to be moderately heritable [5], suggesting that genotype may underlie some of the motivation for participation in such activities.

Most genetic studies of sensation seeking traits have investigated genes encoding components of the dopaminergic pathway due to the purported role of dopamine in behavioural activation and instrumental learning [6], [7]. Variants in the dopamine-4-receptor gene (*DRD4*, 11p.15.5) and its adjacent regulatory region have been associated with personality traits that share a common motivational tendency towards behavioural approach, including, novelty seeking, extraversion, and sensation seeking [8]–[11]. While there are numerous single nucleotide polymorphisms (SNPs) in the promoter region of the gene [12], most have rare minor alleles and are not likely to be informative for studying genetic associations with continuous personality traits [13]; therefore, personality genetics studies on the *DRD4* are often investigated using the −521T/C SNP and a 48-bp repeat in exon 3, which are relatively heterogeneous in most populations. While there is support for the involvement of the *DRD4* in approach-related traits, the results of these studies have been inconsistent [11], [14].

Despite the interest in *DRD4* and high-risk social populations, such as substance abusers and alcoholics [6], there have been few genetic association studies on personality traits in high-risk sport populations [15], [16]. The physiological mechanisms that underlie the motivation to participate in risky antisocial pastimes may be similar to those that attract people to high-risk sports [17]. Sports provide an excellent context to study sensation seeking as participation is quite common in many cultures, there is often a broad spectrum of behavioural options (ranging from cautious to extremely risky), the activities are usually legal and involve non-vulnerable and accessible participants, and patterns of behaviours can be estimated by self-report.

To test the hypothesis that variants in *DRD4* influence sensation seeking traits in athletes, a cross-sectional, single cohort design was employed in a sample of proficient skiers and snowboarders to test for associations between *DRD4* genotypes and sensation seeking phenotypes established using: 1) Zuckerman's measure for impulsive sensation seeking (ImpSS) [18] and 2) the Contextual Sensation Seeking Scale for Skiing and Snowboarding (CSSQ-S, a tool that measures patterns of domain-specific sensation-seeking in skiers/snowboarder) [19]. The subjects were categorized by genotype for a number of polymorphisms in the 5′ up-stream promoter region of *DRD4* and then the sensation seeking phenotypes between each category were compared in order to determine if any of the phenotypes were associated with a specific genotype (or genotypes). Inclusion of polymorphisms was limited to informative, common SNPs (heterozygosity >.20) located in the promoter region of the gene. The *DRD4* promoter contains a high density of SNPs with low linkage disequilibrium [12], and has commonly been studied in association with approach related traits [8]–[11]. This experimental design differs from the typical case: control association study (which initially categorizes by phenotype) and was chosen for the project because the design is commonly employed in behavioural genetics studies [20], [21] when the phenotype is continuous (rather than discrete), as is the case in sensation seeking.

## Methods

### Subjects

Skiers and snowboarders recruited at a winter festival in Whistler, British Columbia, Canada completed two questionnaires (in English) and provided a buccal (cheek) cell swab for DNA preparation (subject demographics are shown in Table 1). A majority (*n* = 363) of the participants described in the current study were included in a previous study, their questionnaire data was used in both studies, but we analyzed different genetic variants [15]. Subjects were between 17 and 49 yrs of age (*n* = 599, mean  = 27.12 years, *SD*  = 6.45) and were pre-screened for ability (inclusion criteria of intermediate or better ability, defined as being capable of skiing/snowboarding an intermediate piste comfortably). The majority of subjects were either skiers (56%) or snowboarders (38%) while the remaining practiced both sports (5%) or were telemarkers (1%). To minimize confounding effects due to differing biogeographical background, we excluded the small number of subjects that did not self-report as being of European descent subjects from the analysis.

**Table 1:** Descriptive statistics for demographic and personality variables.

Variable	Descriptive statistics
Sex	341 males, 258 females
Education	65% post-secondary or higher
Ability	24% intermediate, 31% advanced, 41% expert, 4% other
CSSQ-S†	*M* = 36.34, *SD* = 7.36
ImpSS	*M* = 12.59, *SD* = 3.87
Imp	*M* = 3.78, *SD* = 2.26
SS	*M* = 8.82, *SD* = 2.18

*Note. M* =  mean, SD  =  standard deviation.

† Total participants included in CSSQ-S means differ from sample total (*n* = 578) because skiing/snowboarding ability was less than intermediate or missing.

#### Ethics Statement

We complied with all Canadian tri-council policy ethical standards for the conduct and reporting of research with human subjects. The procedures were further approved by the Clinical Research Ethics Board at the University of British Columbia. Subjects provided written, informed consent, and the ethics board approved the treatment of participants under 19 years of age as emancipated minors able to provide informed consent. All consent was documented and the records were stored in accordance with the university's data storage guidelines in a secure facility.

### Measures

#### Impulsive Sensation Seeking (ImpSS)

Participants completed the 19 item Zuckerman Kuhlman Personality Questionnaire (ZKPQ) ImpSS measure, scored true/false [18]. Data derived from the ImpSS subscale demonstrated acceptable internal reliability (Cronbach alpha  = .80). Sensation seeking as opposed to novelty seeking is the trait more commonly measured in sport populations [1]. The ZKPQ ImpSS was chosen over the more commonly employed Sensation Seeking Scale V [22] to measure global sensation seeking because it contains modern language and lacks potentially confounding items relating to sport. The ImpSS may also be divided into its two factors: impulsivity (Imp, eight items) and sensation seeking (SS, 11 items). The impulsivity factor measures lack of planning and forethought; while the sensation-seeking factor measures the desire to seek out new and thrilling experiences and the willingness to take risks [18]. The two-component scale allows for the consideration of impulsivity and sensation seeking as dissociable traits [23]. If there are significant differences between genotypes and ImpSS, the subscale may be divided into its components (SS and Imp) to investigate whether the association is driven by differences in impulsivity or sensation seeking.

#### Contextual Sensation Seeking Scale for Skiing (CSSQ-S)

Participants completed the 10 item CSSQ-S, anchored on a Likert scale by 1 (strongly disagree) and 5 (strongly agree). Exemplar items from the CSSQ-S include “I like to ski/ride fast” and “I like to go down runs that I have never been down before” [19]. Data derived from the instrument demonstrated high internal consistency (Cronbach alpha  = .87).

### Genotyping

Buccal cell DNA was isolated from cytobrushes (Fisher Scientific, Ottawa, ON, Canada) using an alcohol purification technique [24] and DNA samples were diluted to 20 *n*g DNA/*μ*l. The DNAs were genotyped at Génome Québec Innovation Centre at McGill University, Montréal, Quebec, Canada using the Sequenom iPLEX technique (San Diego, California, USA). The initial goal was to obtain data for five SNPs (*DRD4* −1106T/C (dbSNP rs936460), −906T/C (dbSNP rs3758653), −809G/A (dbSNP rs936461), −616G/C (dbSNP rs747304), −291C/T (dbSNP rs916457)); however, we were unable to obtain data on −616G/C due to amplification problems. We also attempted to obtain −521T/C (dbSNP rs1800955) genotypes for those samples (*n* = 236) that had not previously been analysed [15], but due to failed amplification we were unable to obtain this data.

The 120-base pair tandem duplication was amplified using primers and polymerase chain reaction (PCR) reagents described by Seaman et al. [25] and the touchdown thermal profile described in McCracken et al. [26]. Each 25 *μ*L reaction contained 20 mM Tris-HCl pH 8.4, 50 mM KCl, 1.5 mM MgCl_2_, 0.2 mM dNTP, 0.2 *μ*M of each primer, 5% DMSO, 0.5 U *Taq* polymerase (Invitrogen Corporation, Carlsbad, CA, USA), and 10–20 *n*g DNA template. PCR products were size separated by electrophoresis on 8% PAGE gels run in TBE buffer, stained with SYBR Safe DNA gel stain (Invitrogen, California) and visualized using BIORAD Gel Doc EZ System (Hercules, California). Alleles were characterized by “short” (S; 429 bp – no repeat) versus “long” (L; 549 bp–429 bp +120 bp repeat) amplification products.

### Statistical analyses

An analysis of variance (ANOVA) was used to analyze main effects of the individual genotypes (120 bp duplication (LL + LS *vs*. SS), −1106T/C (TT *vs*. TC *vs*. CC), −906T/C (TT *vs*. TC *vs*. CC), −809G/A (GG *vs*. GA *vs*. AA), −291C/T (CC *vs*. CT *vs*. TT)) and each of the dependent variables (ImpSS, CSSQ-S). As there is no evidence for a dominant or recessive mode of action for any of the SNPs examined, an additive model of inheritance (three factor levels) was applied. The 120-bp tandem duplication was analyzed using both additive and grouped models, as there is evidence that the L allele is dominant [9]. A Bonferroni correction for multiple testing (*i.e*., the five independent genotypes for three dependent variables) was applied to the data, so the threshold for significance was set at an alpha of .003 (.05/15) (for a discussion of this statistial correction method see [27]).

The effects of potential covariates such as sex, age, and ability were investigated before carrying out the analyses. Sensation seeking putatively varies with age and between the sexes [18]; therefore, the relationship between age and sensation seeking was measured using Pearson's correlation, and the effects of sex across sensation-seeking variables were analyzed using an independent sample *t*-test.

## Results

The genotype distributions at the five loci (Table 2) were consistent with Hardy-Weinberg Equilibrium (*p*>.05). The mean marker call rate for SNPs analyzed using Sequenom iPlex was 98±0.33%. Scores derived from the ZKPQ ImpSS and CSSQ-S scales were normally distributed, with no univariate outliers. Sex was entered in the initial analysis as a covariate because there were significant differences between the sexes in both domain-specific and general sensation seeking (CSSQ-S: *t*(576)  = 13.70, *p*<.001; ZKPQ ImpSS: *t*(494)  = 3.20, *p* = .001). Age was related to both CSSQ-S and ImpSS (CSSQ-S *r*(575)  = −.24, *p*<.001; ImpSS *r*(597)  = −.09, *p* = .04), but the relationship between ImpSS and age was too weak to be included as a covariate. Finally, ability was significantly correlated with CSSQ-S, *r*(570)  = .60, *p*<.001 and was included as a covariate for CSSQ-S analyses only.

**Table 2:** Descriptive statistics and ANOVA results for DRD4 promoter polymorphisms.

Marker	dbSNP	Genotype	CSSQ-S			ImpSS		
			*n*	*M*	*SD*	*n*	*M*	*SD*
120-bp repeat	-	LL	258	36.78	7.49	300	12.78	3.84
		LS	123	36.28	6.93	131	12.53	4.00
		SS	13	40.39	4.82	14	13.07	3.65
		LS + SS	136	36.67	6.85	145	12.58	3.96
		*F_A_, F_G_*		1.29,	0.69		0.06,	0.11
		*p_A_ p_G_*		.28,	.41		.94,	0.75
		η_p_^2^		.006,	.002		<.001	
−1106 T/C	rs936460	TT	266	36.45	7.48	281	12.75	3.72
		TC	241	36.04	7.44	256	12.61	4.06
		CC	59	37.31	6.32	62	11.81	3.73
		*F*		1.11			1.47	
		*p*		.33			.23	
		η_p_^2^		.004			.005	
−906 T/C	rs3758653	TT	388	36.30	7.46	409	12.58	3.94
		TC	158	36.12	7.29	169	12.58	3.63
		CC	20	39.50	4.49	21	12.81	4.63
		TC + CC	178	36.50	7.11			
		*F*		0.96^†^			0.01	
		*p*		.33^†^			.99	
		η_p_^2^		.002			<.001	
−809 G/A	rs936461	GG	224	36.16	7.82	235	12.70	3.90
		GA	270	36.46	7.17	285	12.46	3.79
		AA	68	36.84	6.58	75	12.68	4.18
		*F*		0.24			0.18	
		*p*		.79			.84	
		η_p_^2^		.001			.001	
−291 C/T	rs916457	CC	501	36.09	7.45	528	12.52	3.91
		CT	57	37.95	6.10	62	13.00	3.63
		TT	4	42.14	3.98	5	13.40	2.51
		*F*		3.16			0.48	
		*p*		.04			.62	
		η_p_^2^		.011			.002	

*Note*. *F*-statistics, *p*-values, and η_p_^2^ for both additive and grouped models are shown for the 120-bp duplication, other SNPs were analyzed using additive genetic models unless otherwise noted. Sex was included as a covariate for ImpSS, and sex, ability, and age were included as covariates for CSSQ-S analyses. *F*_A_ =  additive model, *F*_G_ =  grouped model, *M* =  mean, SD  =  standard deviation. ^†^Homogeneity of variances was violated; therefore, grouped model was tested. dbSNP numbers are identifiers in the NCBI SNP database (http://www.ncbi.nlm.nih.gov/SNP/). Raw data available upon request.

The initial sample included 599 participants; however, not all DNAs were genotyped successfully for all five loci (the 120-bp duplication was especially problematic), so the final sample sizes for the genetic analysis ranged from *n* = 445 to 599. The mean values for the phenotypes (ImpSS and CSSQ-S) did not vary between the full sample (*n* = 599), and the sample that included genotypes for the 120-bp duplication (*n* = 445; *p*>.05). Univariate analyses comparing ImpSS (with sex as a covariate) between genotypes at each loci revealed no significant associations, the results for the impulsivity and sensation seeking scales when analyzed separately were also not significant (data not shown). Similarly, there were no significant associations (at an alpha of .003) between domain-specific sensation seeking, CSSQ-S (with ability, age, and sex as covariates) and any of the polymorphisms tested (see Table 2). The −291 C/T SNP was marginally significant (*p* = .04), though not after consideration of multiple testing.

## Discussion

The individual effects between five *DRD4* promoter polymorphisms and two measures of sensation seeking were investigated in a sample of proficient skiers and snowboarders. There were no significant associations between either the CSSQ-S (the domain-specific sensation-seeking measure) scores or the ZKPQ ImpSS scores and genotype for any of the *DRD4* promoter variants tested.

The *DRD4* has been implicated in numerous association studies of traits relating to behavioural approach and externalizing disorders [11], [28]. The 120-bp tandem duplication has been of particular interest as it contains binding sequences for transcription factors [25] and in some studies, the long (240-bp) allele has reduced transcriptional activity compared to the short allele, which could result in fewer D4 receptors and ultimately affect levels of dopamine in the synapse [29]. Phenotypic data for the short and long alleles of the tandem duplication are inconsistent in the literature. The long version of the tandem duplication has been associated with attention deficit hyperactivity disorder (ADHD) and schizophrenia, conditions that are sometimes characterized by impulsivity [26], [30]; whereas, other studies have reported associations between the short allele and ADHD and/or high impulsivity [9], [31]. Two recent meta-analyses conclude that there is no association between the 120-bp tandem and ADHD [28], [32]. In the current study, there were no significant differences in self-reported impulsive sensation seeking (nor for impulsivity and sensation seeking measured separately) between the 120-bp duplication alleles.

Despite their location in the regulatory region of an important neurotransmitter receptor gene, there are few personality genetic studies that include the four SNPs that we present data for (−291C/T, −809G/A, −906T/C, and −1106T/C). The same four SNPs were investigated in association with schizophrenia in two Japanese populations [12], [33], while Laksy-Su et al. (2007) and Oades et al. (2008) looked for a relationship between the −906T/C genotypes and ADHD in white Europeans (the former also included the −291C/T genotypes in their investigation) [34], [35], and Derringer et al. (2010) and Heck et al. (2009) investigated −906T/C in novelty and sensation seeking [36], [37]. Consistent with our results, none of the aforementioned studies reported significant associations between *DRD4* promoter alleles and the phenotypes being investigated.

This is one of the first studies to investigate *DRD4* variants in a specialized cohort of athletes. One advantage of investigating genetic associations in cohorts that have distinctive, shared characteristics (e.g. gambling problems or athletic behaviours) is that domain-specific tools can be used to measure phenotypes [38]. Thomson et al. (2013) reported a significant association between the *DRD4* −521T/C variant and domain-specific sensation seeking in skiers and snowboarders, but there was no association with a broader measure of sensation seeking [15]. Similarly, studies on individuals exhibiting externalizing behaviours (e.g., alcohol misuse) have found genetic associations with domain-specific measures but not when comparing genotypes across groups defined by broad diagnostic criteria or personality traits [39]. We included an athletic cohort that met the criteria for employing a narrow, domain-specific measure (the CSSQ-S) that has high reliability and showed strong cirterion validity [19]. Narrower traits (or facets) can predict variance in specific behaviours not accounted for by more general traits [40] like sensation seeking.

While there are benefits to studying homogeneous samples sharing distinctive characteristics (e.g., reducing extraneous variability), there are some limitations that should be considered before drawing broad conclusions from the results. In this study, the cohort was limited to: 1) experienced skiers and snowboarders (to ensure that respondents had sufficient ability to carry out the sport behaviours described in the CSSQ-S) and, 2) individuals of Northern European ancestry (to minimize effects of population stratification; see [27]). As such, the results may have limited generalizability. Exclusion of beginner athletes also impacted the number of potential subjects. The total sample (*n* = 599) was sufficient to achieve adequate power (∼.8) based on small effect sizes (η_p_^2^ = .01 to .02) typical of SNP associations at an alpha of .01 [41]. While there is a possibility for Type II error, the effect sizes for most analyses were small (η_p_^2^<.005) suggesting that larger samples would probably find similarly non-significant results assuming comparable estimates of population parameters. Limited by power, we restricted our analysis to include highly heterogenic loci but acknowledge that it would be worthwhile to investigate the effects between all polymorphisms (including those with rare minor alleles) in the 1.2 KB region of the *DRD4* promoter.

The initial goal of this study was to genotype all of the polymorphisms in the promoter region with high heterogeneity; however, two (−521T/C and −616C/G) failed to optimize and, two were deletion polymorphisms (−603del/T and −1217G/del describe in [12]), which could not be assayed by multiplex genotyping technique used in this study. Absence of data for the deletion polymorphisms likely has little impact on the strength of the conclusions as the loci are reportedly in disequilibrium (e.g., alleles at −603del/T and −1217G/del are in moderate-to-strong linkage disequilibrium with alleles at −1106T/C [12]), which allow the alleles that were assayed to serve as proxy for other alleles on the same haplotypes.

In summary, five polymorphisms in the *DRD4* promoter were tested for associations with sensation seeking in a sample of skiers and snowboarders. There were no significant associations between genotypes at these loci and sensation-seeking measures. These findings are the first to be reported in athletes and for the most part are in line with results from previous studies done in clinical and general populations, which reported no associations between approach-related traits and other *DRD4* promoter variants other than the −521T/C.
